# Proinflammatory-activated trigeminal satellite cells promote neuronal sensitization: relevance for migraine pathology

**DOI:** 10.1186/1744-8069-5-43

**Published:** 2009-08-06

**Authors:** Alessandro Capuano, Alice De Corato, Lucia Lisi, Giuseppe Tringali, Pierluigi Navarra, Cinzia Dello Russo

**Affiliations:** 1 Institute of Pharmacology, Catholic University School of Medicine, L.go F Vito Rome, Italy

## Abstract

**Background:**

Migraine is a complex, chronic, painful, neurovascular disorder characterized by episodic activation of the trigeminal system. Increased levels of calcitonin gene-related peptide (CGRP) are found at different levels during migraine attacks. Interestingly, CGRP is also released within the trigeminal ganglia suggesting possible local effects on satellite cells, a specialized type of glia that ensheaths trigeminal neurons. CGRP was shown to enhance satellite-cell production of interleukin 1β (IL-1β), while trigeminal neurons express an activity-dependent production of nitric oxide (NO). Thus, in the present study we tested the hypothesis that IL-1β and NO induce trigeminal satellite cell activation, and that once activated these cells can influence neuronal responses.

**Results:**

Primary cultures of rat trigeminal satellite cells isolated from neuronal cultures were characterized in vitro. Cyclooxygenase (COX) expression and activity were taken as a marker of glial pro-inflammatory activation. Most of the experiments were carried out to characterize satellite cell responses to the two different pro-inflammatory stimuli. Subsequently, medium harvested from activated satellite cells was used to test possible modulatory effects of glial factors on trigeminal neuronal activity. IL-1β and the NO donor diethylenetriamine/nitric oxide (DETA/NO) elevated PGE2 release by satellite cells. The stimulatory effect of IL-1β was mediated mainly by upregulation of the inducible form of COX enzyme (COX2), while NO increased the constitutive COX activity. Regardless of the activator used, it is relevant that short exposures of trigeminal satellite cells to both activators induced modifications within the cells which led to significant PGE2 production after removal of the pro-inflammatory stimuli. This effect allowed us to harvest medium from activated satellite cells (so-called 'conditioned medium') that did not contain any stimulus, and thus test the effects of glial factors on neuronal activation. Conditioned medium from satellite cells activated by either IL-1β or NO augmented the evoked release of CGRP by trigeminal neurons.

**Conclusion:**

These findings indicate that satellite cells contribute to migraine-related neurochemical events and are induced to do so by autocrine/paracrine stimuli (such as IL-1β and NO). The responsiveness of IL-1β to CGRP creates the potential for a positive feedback loop and, thus, a plurality of targets for therapeutic intervention in migraine.

## Background

Migraine is a complex, chronic, painful, neurovascular disorder characterized by episodic activation of the trigeminal system; in particular, trigeminal ganglia play a pivotal role in initiating and maintaining pain [1,2]. Cell bodies of trigeminal neurons extend their axonal projections to the brainstem nuclei, which mediate the transmission of nociceptive information to the higher brain centers where pain is perceived. On the other side, peripheral nociceptive fibers arising from trigeminal neurons innervate cerebral vessels as well as vessels within the dura and pia mater, i.e. the cranial structures likely responsible for pain in migraine [3]. Several neuropeptides have been identified in the trigeminal ganglia, including substance P, neurokinin A, and calcitonin gene-related peptide (CGRP). The latter is thought to be the main neuromediator of trigeminal signaling, particularly in headache [4]. In fact, increased levels of CGRP are found in the jugular vein during migraine attacks [5], and an increase in CGRP release occurs during peripheral sensitization of meningeal nociceptors, which is responsible for hyperalgesia and allodynia often associated with migraine [6].

CGRP is also released in a paracrine/autocrine manner within the trigeminal ganglia [7,8], suggesting a role as a local modulator. Moreover, sustained activation of sensory neurons is associated with increased nitric oxide (NO) production from neuronal NO synthase (nNOS) [9-11]. Because of its physico-chemical properties, NO can rapidly diffuse among the cells and modulate intracellular functions, as well as extracellular events such as neurotransmitter receptor activation. Interestingly, trigeminal neurons are surrounded by glial cells, referred to as satellite cells; both cell types form functional units within the tissue [12]. Satellite cells appear to respond to neuronal activity with the secretion of factors that can modulate such activity [13]. In particular, intraganglionic CGRP induces satellite cells to produce cytokines [14], among which IL-1β, along with other inflammatory mediators such as NO [7]. At the present, the impact of these inflammatory mediators on glial functions is not fully clarified.

In this study, we tested the hypothesis that IL-1β and NO can induce trigeminal satellite cell activation, and that once activated these cells may influence neuronal responses. Thus, we developed primary cultures of rat trigeminal satellite cells and characterized their activation in vitro. Subsequently, medium harvested from activated satellite cells was used to test possible modulatory effects of glial factors on trigeminal neuronal activity. This experimental model allowed us to study components of a cyclical satellite-neuron interaction, beginning at the point of inflammatory stimuli. We found that both IL-1β and NO stimulated an increased production of PGE2, although the underlying activations of COX were mechanistically distinct. Once such activation was characterized, we used these satellite cells to generate conditioned media that were tested on primary cultures of trigeminal neurons to investigate the possible influence of glial factors on neuronal CGRP release. Conditioned medium from activated satellite cells augmented CGRP release evoked by a standard depolarizing agent. Our data clearly suggest that satellite cells, besides providing a mechanical and metabolic support to neurons, directly modulate their functions. A patho-physiological feed-forward loop between satellite cells and neurons may thus contribute to migraine. This view provides the opportunity for multiple -and, perhaps, combinatorial -therapeutic strategies because interrupting either the glial or the neuronal end of this interaction should break the cycle and effect attenuation of migraine duration and/or intensity.

## Results

### Morphological and functional characterization of rat trigeminal satellite cells in vitro

**Figure 1:**
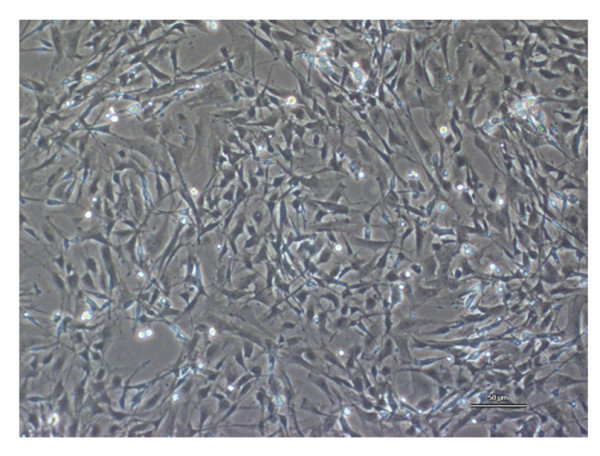
**Primary cultures of trigeminal satellite cells**. Morphology of a primary culture of rat satellite cells was observed under phase-contrast microscopy. The predominant phenotype consists of small and dark cells with an elongated shape. (10× – 50 μm scale bar)

**Figure 2:**
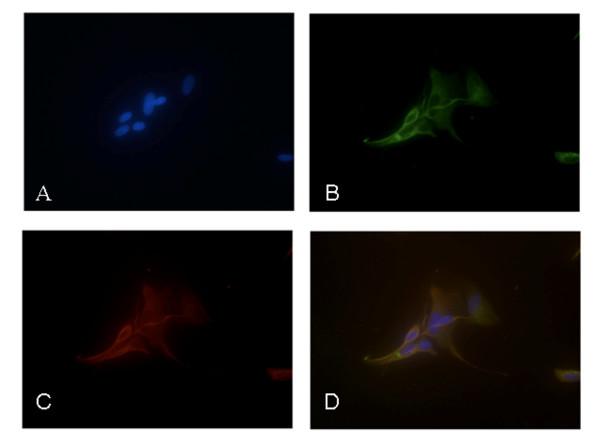
**Immunostaining of primary cultures of trigeminal satellite cells**. Cells were stained with the nuclear dye DAPI (**A**). Immunocytofluorescence was performed with antibodies against the glial fibrillary acidic protein (GFAP) (**B**) and glutamine synthase (GS) (**C**). The same field is shown in all three panels, and a merged representation of the triple-staining is presented in panel **D**. (40× – 50 μm scale bar)

**Figure 3:**
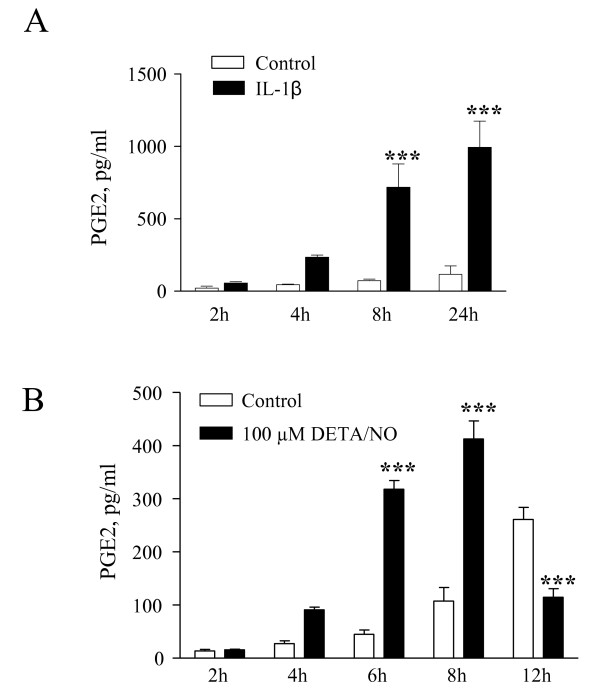
**Time-dependent increase of PGE2 release from rat trigeminal satellite cells**. Cells were incubated with or without tested stimuli 10 ng/ml IL-1β (**A**) or 100 μM DETA/NO (**B**). PGE2 secretion was evaluated at different time points by RIA (up to 24 h for IL-1β). Data are expressed as pg/ml of PGE2, and are means ± SEM of n = 6 replicates for each experimental group. *** *p *< 0.001 *vs*. control (two-way ANOVA followed by Bonferroni's post-hoc test).

Rat trigeminal satellite cells were isolated from dissociated suspensions of trigeminal ganglia, exploiting their tendency to firmly adhere to the bottom of uncoated culture flasks [15]. Cells were subcultured twice and further expanded in vitro, then used for functional experiments once they reached almost complete confluence (Figure 1). Two specific markers of glial cells, the glial fibrillary acidic protein (GFAP) and the glutamine synthase (GS), were expressed in trigeminal satellite cells. As shown in Figure 2, it is possible to distinguish two types of satellite glial cells via morphological and immunological phenotypes. The most abundant cells (65%) are strongly positive for GFAP and GS and have a small cell body with an elongated shape; roughly 35% of the total cells stained weakly for the two glial markers and have an astrocyte-like morphology. In preliminary experiments we studied the effects of different proinflammatory stimuli on satellite cell activation, taking the levels of PGE2 released in the incubation media as a marker of cellular inflammatory response. Under basal conditions primary cultures of trigeminal satellite cells release sizable amounts of PGE2 in a time-dependent manner (Figure 3). IL-1β (10 ng/ml) significantly increased PGE2 release from 8 h onward (Figure 3A). Other proinflammatory cytokines, such as IFNγ (10 UI/ml) and TNFα (10 ng/ml), produced no effect on PGE2 after 24 h treatments (data not shown). In preliminary experiments, we found that the NO donor DETA/NO increased PGE2 release after 6 h, with a peak response produced by 100 μM DETA/NO; therefore, this concentration was used in all subsequent experiments. DETA/NO significantly increased PGE2 release after 6 and 8 h, while a significant inhibitory effect was observed at 12 h of incubation (Fig. 3B). The levels of PGE2 measured after 8 h of exposure to DETA/NO were lower than those induced by an 8-h treatment with IL-1β, suggesting that different molecular mechanisms are recruited by the two stimuli.

**Figure 4:**
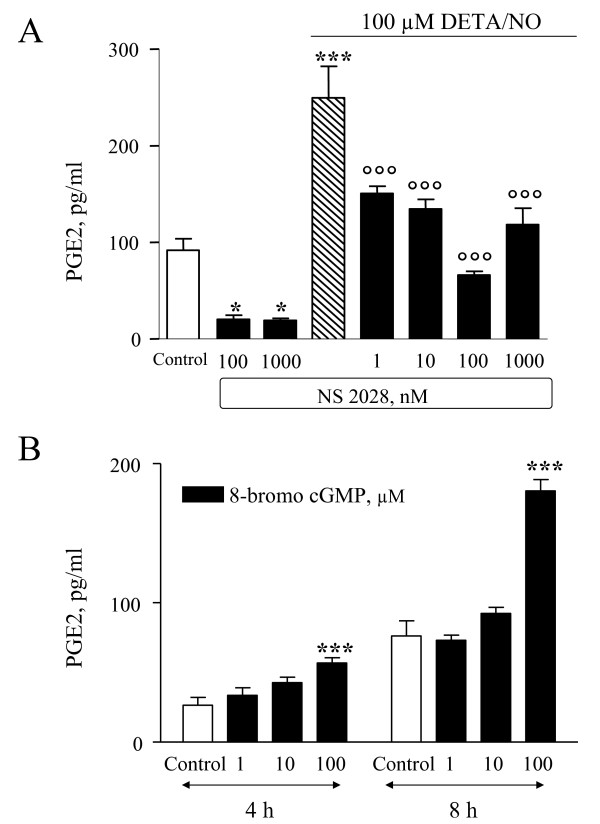
**Involvement of cGMP signaling in the effects of DETA/NO on PGE2 release**. **A**: Cells were incubated for 6 h with or without 100 μM DETA/NO. The guanylate cyclase inhibitor NS2028 was included as indicated. Data are expressed as pg/ml of PGE2 and are means ± SEM of n = 6 for each experimental group. * *p *< 0.05, *** *p *< 0.001 *vs *control; °°° *p *< 0.001 *vs *DETA/NO alone. **B**: Cells were incubated with the indicated concentrations of the stable and cell-permeable analog of cGMP, 8-Br-cGMP. Data are expressed as pg/ml of PGE2, as mean ± SEM of n = 6 replicates for each experimental group. *** *p *< 0.001 *vs*. controls.

Some effects of NO are mediated by the activation of the soluble guanylyl cyclase (sGC) and the subsequent increase in intracellular cGMP levels. In the range 1 nM-1 μM, the sGC inhibitor NS2028 significantly reduced the stimulatory effect of DETA/NO (Fig. 4A). At higher concentrations, NS2028 even inhibited basal PGE2 release. Consistent with these data, we found that the stable and cell-permeable cGMP analog 8-Br-cGMP increased PGE2 release after 4-h and 8-h treatments, with significant effects observed at 100 μM (Figure 4B). Taken together, these results indicate that the stimulatory effects of DETA/NO are mediated at least in part by increased levels of cGMP.

### Effects of pro-inflammatory stimuli on cyclooxygenase expression

**Figure 5:**
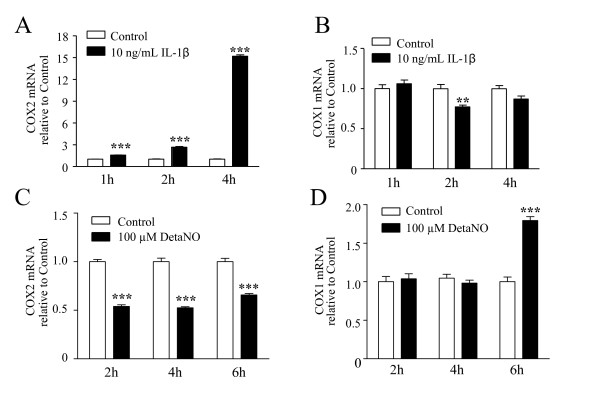
**IL-1β and DETA/NO differentially induce COX1 and COX2 expression in rat trigeminal satellite cells**. Satellite cells were incubated with 10 ng/ml IL-1β (**A **and **B**) or 100 μM DETA/NO (**C **and **D**) for the indicated times, then total RNA was extracted. Expression of COX2 (**A **and **C**) or COX1 (**B **and **D**) was evaluated by real-time (Q) RT-PCR using the comparative quantitation analysis. Data are expressed as the signal of mRNA from treated cultures relative to that from untreated controls (± SEM; *n *= 3); data were compiled from two independent experiments. ** *p *< 0.01, *** *p *< 0.001 *vs*. controls.

**Figure 6:**
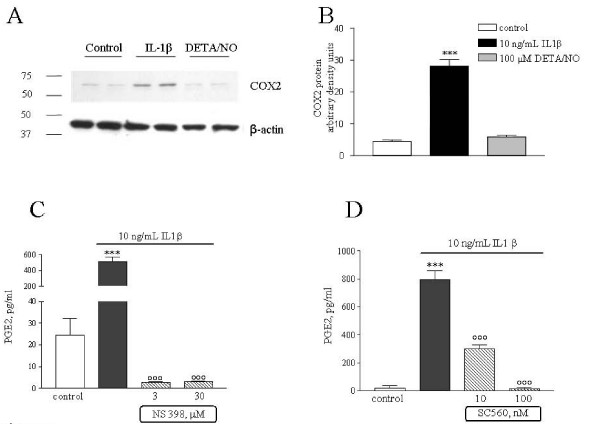
**COX-2 protein expression induced by IL-1β in trigeminal satellite cells**. (**A**) Whole-cell lysates were prepared from control satellite cells or cell treated with either IL-1β or DETA/NO for 4 h (2 replicates per each treatment), and equal amounts of protein were analyzed by western blot analysis for COX-2. The same blots were subsequently probed for β-actin. (**B**) Quantitation of densitometry wherein COX-2 values are reflected relative to those for β-actin. Data are expressed as mean ± SEM of *n *= 2 replicates for each group, each assayed in triplicates. *** *p *< 0.001 *vs*. controls. Satellite cells were incubated in presence of 10 ng/ml IL-1β together with the selective COX2 inhibitor, NS398 (**C**) or the selective COX1 inhibitor, SC560 (**D**) for 8 h. Data are expressed as pg/ml of PGE2, as mean ± SEM of n = 5 replicates for each experimental group. *** *p *< 0.001 *vs*. controls; °°° *p *< 0.001 *vs *IL-1β alone.

Under resting conditions, cells of the immune-inflammatory lineages mainly produce prostaglandins via the activity of the constitutive COX1, whereas inflammatory situations evoke inductions of COX2. IL-1β significantly increased expression of COX2 in trigeminal satellite cells (Figure 5A). After a 4-h treatment, IL-1β evoked a 15-fold increase in the levels of COX2 mRNA without influencing COX1 mRNA levels (Figure 5B). DETA/NO appeared to have a distinct mechanism of action; its stimulatory effect on PGE2 release was associated with only a modest increase in COX1 mRNA levels observed after 6 h incubation (Figure 5D), while COX2 mRNA levels were reduced (Figure 5C). Consistently, western blot analysis showed increased amount of COX2 protein in the lysates of cells treated with IL-1β for 4 h, while no effect was observed in cells treated with DETA/NO (Figure 6A). In figure 6B a densitometry analysis of the blot is reported; values for COX2 protein levels are normalized by the density values relative to the correspondent β-actin, as explained in detail in the Methods. Furthermore, the stimulatory effect of IL-1β on PGE2 release was completely antagonized by the selective COX2 inhibitor NS398 given in the range of 3–30 μM. The inhibitory effect of NS398 was already maximal at 3 μM, a concentration equal to the IC50 calculated for the purified enzyme (Figure 6C). In contrast, the selective COX1 inhibitor SC650, at 10 nM (~IC50), only partially even though still significantly reduced PGE2 production increased by IL-1β (Figure 6D). Both 30 μM NS398 and 100 nM SC560 significantly reduced basal PGE2 release, thus suggesting that both isoforms contribute to prostaglandins production under basal condition. However, data obtained using COX inhibitors in presence of IL-1β support the notion that COX2 upregulation and activity mainly mediates the stimulatory effects of IL-1β on PGE2.

### Effects of satellite cell conditioned media on neuronal activation

**Figure 7:**
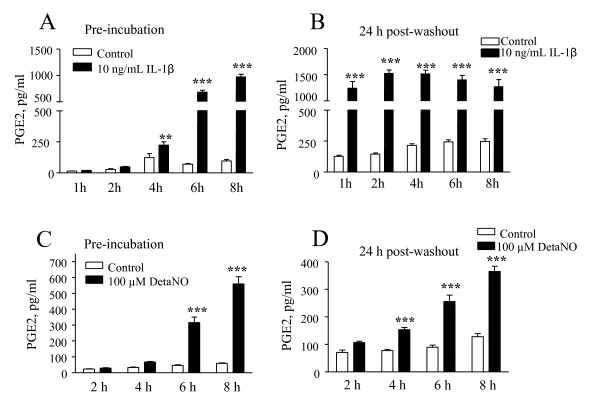
**Persistent effects of pro-inflammatory stimuli on satellite cell activation**. Satellite cells were incubated for different times in presence of 10 ng/ml IL-1β (**A**) or 100 μM DETA/NO (**C**), and the levels of PGE2 in the medium were assayed by RIA. Then the cultures were washed and further incubated in fresh medium for an another 24 h (see Methods for details). PGE2 that had accumulated during this 24 h incubation was assayed in the various treatment groups, i.e., different times of initial treatment in the presence of IL-1β (**B**) or DETA/NO (**D**). Data are expressed as pg/ml of PGE2, as mean ± SEM of n = 6 replicates for each experimental group. **, *p *< 0.01; *** *p *< 0.001 *vs*. controls

Satellite cells were incubated for different times with IL-1β or DETA/NO to generate conditioned medium, subsequently used to test the effects of glial mediators on neuronal CGRP release (see Methods). Similarly to the experiments described in Figure 3, IL-1β increased PGE2 release in a time dependent manner and statistically significance reached from 4 h onward (Figure 7A). The effects of IL-1β were long-lasting, in fact pre-exposure of trigeminal satellite cells to the cytokine elevated the levels of PGE2 in the following 24 h incubation in plain medium, regardless of the length of such preincubation (Figure 7B). Trigeminal satellite cells were exposed to DETA/NO for different times (2–8 h) and further incubated in plain medium for 24 h after DETA/NO removal. As shown in Figure 7C, significant increases in PGE2 release were observed after 6 and 8 h incubation, while an even shorter pre-exposure to DETA/NO (4 h) increased PGE2 release in the following 24 h incubation (Figure 7D). Higher levels of PGE2 were measured after 6 and 8 h pre-incubation with DETA/NO (Figure 7D). Based upon these results conditioned media from satellite cells activated for 4 h with IL-1β or 6 h with DETA/NO were used on neuronal cultures.

**Figure 8:**
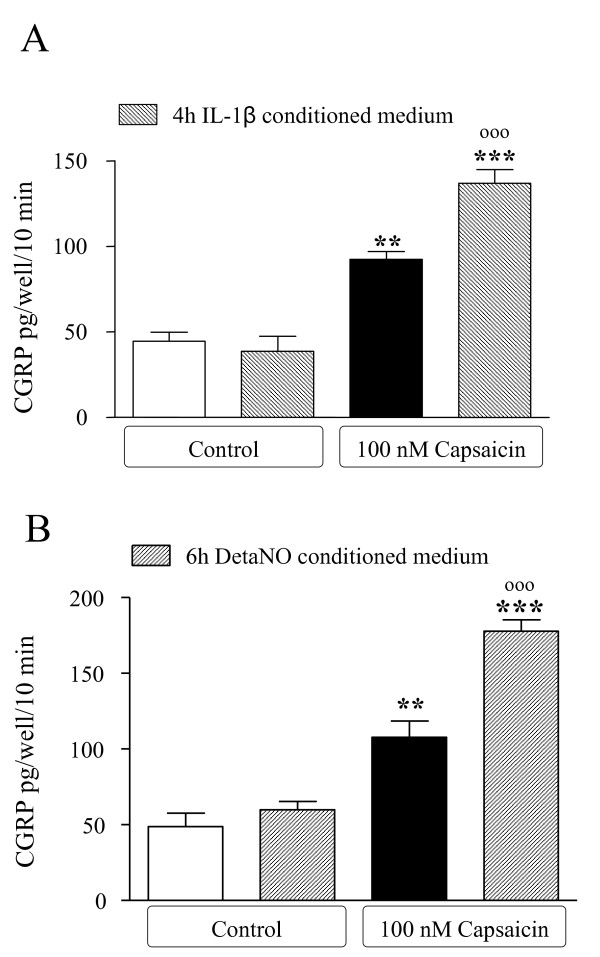
**Conditioned media from activated satellite cells enhances CGRP release from trigeminal neurons**. Satellite cells were exposed to IL-1β for 4 h (**A**) or DETA/NO for 6 h (**B**), then washed and incubated in fresh medium for another 24 h, as described in Fig. 7. Aliquots of this second cycle of conditioned medium (lacking the initial activating stimuli) were applied to trigeminal neurons at a 1:1 dilution, and CGRP was measured. After 30 min, capsaicin (100 nM) was applied to evoke secretion of CGRP in trigeminal neurons that had been pre-exposed to conditioned medium or not. Data are expressed as pg/well/10 min of CGRP, as mean ± SEM of *n *= 6 replicates for each experimental group. ***, *p *< 0.001 *vs*. controls; °°° *p *< 0.001 *vs*. capsaicin alone.

Pre-exposure of trigeminal neurons for 30 min to IL-1β conditioned medium (diluted to 50%) significantly increased the release of neuronal CGRP evoked by 10 min stimulation with 100 nM capsaicin (Figure 8A) [15]. In a similar manner, conditioned media derived by satellite cells activated with DETA/NO significantly enhance the stimulatory effects of capsaicin (Figure 8B).

## Discussion

In the present study we have characterized rat primary cultures of trigeminal satellite cells, prepared after neuronal removal, both under basal condition and after exposure to different pro-inflammatory stimuli. Particularly, we found that both the inflammatory cytokine IL-1β and the gaseous mediator NO increase PGE2 production by trigeminal satellite cells. While the effect of IL-1β was mainly mediated by upregulation of the inducible form of COX enzyme (COX2), NO seemed to act by increasing COX activity without any relevant modification of the expression of these enzymes. Regardless of the activator used, it is relevant that short exposures of trigeminal satellite cells to both activators induced modifications within the cells which led to significant PGE2 production after removal of the pro-inflammatory stimuli. This effect was used to generate conditioned media from activated satellite cells that did not contain any activators; such media were used to test the effects of glial factors on neuronal activation. In fact, within the sensory ganglia neurons are unsheathed by several glial cells, forming morphological and functional units [16], and neuronal-glial interactions have been recently described in vivo, suggesting that satellite cells can modulate neuronal activity in several manners [17,18]. Satellite cells are thought to actively participate to pain transmission [12] since they regulate the local ganglion environment by removing the excess of ions and neurotrasmitters [16]. Moreover, several receptors, including those for IL1β, have been detected on the surface of satellite cells under basal conditions [19], while other receptors can be expressed during pathological processes among which those for inflammatory cytokines [20]. Thus, it may be hypothesized that trigeminal satellite cells are activated during neurogenic inflammation, and promote and maintain activation of trigeminal neurons by releasing pro-inflammatory mediators. One of the most important pathways in the development of neuronal sensitization is the activation of COX enzymes leading to increased prostaglandin production. These mediators do not interfere directly with neuronal neurotransmitter release, such as CGRP release, but can reduce the firing threshold of sensory neurons in response to other depolarizing agents [21,22]. In light of this evidence, we focused our studies on COX expression and activity, particularly considering the important role of prostaglandins in mediating neuronal sensitization [23]. Moreover, our experimental model allowed us to study possible neuronal-glial interactions in a controlled simplified paradigm.

IL-1β increased in a time dependent manner the release of PGE2, which paralleled significant induction of COX2 mRNA expression and protein synthesis (Figures 5 and 6). Data obtained with selective pharmacological inhibitors of COX activity, showed the involvement of both enzyme in mediating PGE2 release by satellite cells. However, in response to IL-1β challenge COX2 activity appears to be predominant. The signaling pathways underlying the effects of IL-1β are well characterized, and include the activation of membrane bound IL-1 receptors that triggers a complex cascade of phosphorylations leading to downstream activation of Inhibitor-kB kinase (IKK) and mitogen activated protein kinases (MAPKs), as well as of transcription factors such as NF-kB [24]. These kinases and activated NFkB are known inducers of COX2 expression [25]. Even though we did not thoroughly explore the molecular mechanisms underlying the stimulatory effects of IL-1β, we may reasonably postulate the involvement of such mechanisms, in the same manner as they occur in several other cell types expressing IL-1β receptors. Particularly, IL-1β-dependent COX2 induction and PGE2 production have been described in glial cells in several neurological disorders, such as multiple sclerosis and Alzheimer's disease [26]. Reactive glial cells actively participate in central sensitization during chronic pain, and their activation mediates pain hypersensitivity [27,28]. The role of activated glial cells and their interactions with neurons in the transmission of pain sensation has been extensively studied in specific brain areas and in the spinal cord during pain disorders [28,29]. Conversely, the relevance of glial activation within the peripheral sensory ganglia, and their contribution in mediating pain signaling, are not yet fully clarified. Several experimental data suggest that proinflammatory cytokines such as IL-1β are released in peripheral sites of inflammation from immune cells and may affect neuronal excitability in a paracrine/endocrine manner [20,30-32]. Indeed, IL-1β is able by itself to promote neuronal discharge; the cytokine also sensitizes neurons to other stimuli lowering the neuronal firing threshold, via the modulation of different ion fluxes (e.g. Na^+^, and K^+^) [33-35]. On the other hand, IL-1β contributes to the amplification of inflammatory processes promoting the production of secondary modulators such as NO, bradykinin and prostaglandins, which can in turn exert modulatory actions on neuronal activity [9,36]. Recently, it has been shown that CGRP is also released within the trigeminal ganglia, possibly enhancing local inflammation [7,8]. In fact, it has been demonstrated that neuronal CGRP can increase the expression of several pro-inflammatory genes among which IL-1β [7]. Thus, we hypothesized that these pro-inflammatory mediators may act locally to increase both glial and neuronal responses, thereby sustaining the activation of the trigeminal system.

We have also shown that satellite glial cells can be activated in vitro by short term exposure to the NO donor, DETA/NO. This drug slowly releases small amounts of NO, with a half-life of 20 h, thus better mimicking the in vivo condition [37]. In fact, in dorsal root ganglia it has been shown that depolarizing stimuli enhancing intracellular Ca^++ ^concentrations, such as capsaicin and bradykinin, can increase NO production from clusters of nNOS positive sensory neurons [10,11,38]. Interestingly, these neurons were surrounded by cGMP positive satellite cells, suggesting that NO may diffuse within the glia and activate sGC [39,40]. Apart from the fact that a pivotal role for NO among all pain mediators has been clearly demonstrated in migraine pathology [41-43], little is known about its effects on trigeminal satellite cell functions. In our model, DETA/NO induced a transient increase in PGE2 production, an effect in part mediated by activation of sGC. In fact, the sGC inhibitor NS2028 reduced the stimulatory effects of DETA/NO, and the stable cGMP analog, 8-bromo-cGMP, mimicked DETA/NO effects. Moreover, NS2028 given alone reduced the basal release of PGE2, indicating that cGMP contributes to the regulation of COX activity. However, we cannot rule out other possible mechanisms, such as the modulation of COX activity via post-translational modifications of the enzyme. These mechanisms have been described in several cell cultures, but the NO-COX interaction has been reported to produce different and even opposite effects [44]. NO-dependent activation of COX can be related to the antioxidant properties of NO, or it may be mediated by the generation of superoxide or peroxynitrite radicals. In this framework, it is important to consider the source of NO release, whether constitutive or inducible NOS activity, and thus the redox status of resting or *inflamed *cells [44]. Nanomolar concentrations of NO in the system, usually associated to nNOS activity, act as negative modulator of NFkB, inducing down regulation of COX2 expression, whereas micromolar concentrations of NO (via iNOS) upregulate COX2 [45]. In our model, we did observe a reduction of COX2 mRNA levels after exposure to DETA/NO, which produces a controlled release of low amount of NO thus mimicking the production of nanomolar concentrations of NO by nNOS activity [45]. However, the overall effect of NO on PGE2 production is a stimulation lasting up to 8 h incubation time, which suggests the involvement of alternative mechanisms for NO, as for example a direct stimulatory effects on COX activity or the induction of COX1.

These results support the hypothesis that glial cells can be activated by changes in neuronal activity, and further release glial factors that modulate neuronal responses. Thus, to test this hypothesis we studied the effects of IL1β- or NO- conditioned media on CGRP release from primary cultures of trigeminal neurons. We have previously shown that the depolarizing agent capsaicin increases CGRP release from trigeminal neurons; this paradigm can be used as a marker of neuronal activity [15]. Conditioned media did not influence basal CGRP release, but were able to significantly increase the stimulatory effects of capsaicin. Trigeminal neurons were pre-exposed to conditioned media derived from both IL-1β and NO activated satellite cells for 30 min, and then stimulated with 100 nM capsaicin for 10 min. Since both stimuli were no longer contained in the conditioned media, the potentiating action observed on neurons is due to factors released by satellite cells under IL-1 β or NO challenge.

## Conclusion

**Figure 9:**
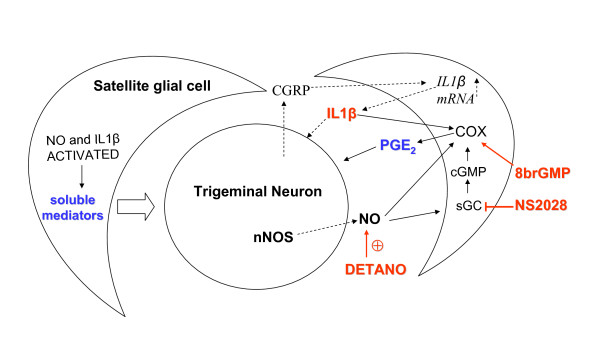
**Mechanisms underlying neuron-glia interaction in trigeminal ganglia**. Satellite glial cells ensheath trigeminal neurons constituting a functional unit into the trigeminal ganglion, as represented in the schematic. It has been demonstrated (dashed arrows) that trigeminal neurons release CGRP and NO within the ganglia during activation. CGRP was shown to increase IL1β secretion by trigeminal satellite cells (dashed arrows). Thus, IL-1β and the NO donor DETA/NO (red) were tested on satellite cells. DETA/NO better mimics neuronal NO release generated by nNOS. Both stimuli increase glial COX activity, even though via different molecular mechanisms (arrows). Conditioned media harvested from glial cells challenged with both stimuli promote neuronal sensitization. Compounds exogenously added to trigeminal satellite cells in different experimental paradigms are indicated in red.

In the present paper, we have characterized primary cultures of rat trigeminal satellite cells and their activation in vitro by two different proinflammatory stimuli relevant to migraine pathology. As shown in the schematic (Figure 9), both stimuli increase COX activity in satellite cells although with different molecular mechanisms. Moreover, conditioned media from activated glial cells induce neuronal sensitization to depolarization. One possible mediator of neuronal sensitization is PGE2, but other factors can contribute to such neuronal response. Interestingly, modifications of neuronal responses occurred after short-term exposure to glial-derived factors, which strongly underlies the importance of glial-neuronal interactions during trigeminal activation. In conclusion, satellite cells are actively involved in the modulation of trigeminal neuronal activity, can amplify and sustain inflammatory processes within the ganglia. These glial effects may be important in the pathophysiology of migraine, and thus satellite cells can be envisioned a new target in pain treatment.

## Methods

### Drugs

Diethylenetriamine/nitric oxide (DETA/NO) was purchased by Sigma-Aldrich (St. Louis. MO), dissolved in distilled water and 10 mM aliquots were made. The selective inhibitor of nitric oxide (NO)-sensitive soluble guanylyl cyclase, NS 2028, was purchased by Alexis Biochemicals (Lausen, Switzerland), dissolved in DMSO at 1 mM. 8-Bromo-cGMP was purchased from Tocris Bioscience (Bristol, UK) as sodium salt, dissolved in distilled water and 10 mM aliquots were made. All drug aliquots were stored at -35°C. Human recombinant IL-1β (Endogen, Pierce Biotechnology, Rockford, IL) was dissolved in distilled water at the concentration of 40 μg/ml and stored in aliquots at -80°C. All working dilutions were made using cell culture medium. None of the tested compounds interfered with radioimmunoassays.

### Cell Cultures

Neuronal and satellite cell cultures from trigeminal ganglia were prepared from 6- to 7-day-old Wistar rats. The use of animals for this experimental work was previously approved by the Italian Ministry of Health (licensed authorization to P. Navarra). Briefly, both trigeminal ganglia were aseptically removed. Tissues were collected in a Petri dish containing 3–5 ml of ice-cold phosphate buffer saline without Ca^2+ ^and Mg^2+ ^(PBS w/o; Sigma), supplemented with antibiotics (100 IU/ml penicillin and 100 μg/ml streptomycin; Sigma) and D-glucose (6 g/l). Tissues were then digested in 5 mg/ml collagenase (Biochrom AG, Berlin, Germany) for 20 min at 37°C, followed by 10 min incubation with 0.125% trypsin (Biochrom) including DNAse I (Sigma) treatment in the last 5 min of incubation. At the end of the incubation time, the enzyme solution was replaced with 5 ml Ham's F12 medium (Biochrom), containing 10% heat-inactivated endotoxin-free foetal calf serum (FCS; GIBCO, Invitrogen Corporation, Paisley, Scotland) and antibiotics (complete culture medium). Cells were mechanically dissociated using a Pasteur pipette, plated on a 25 cm^2 ^flask, and incubated at 37°C in a humidified atmosphere containing 5% CO_2 _for 2–3 h (*pre-plating*). This step allowed us to separate satellite cells from neurons using their differential adhesion properties. Satellite cells retain several characteristics of glial cells, such as the ability to firmly adhere to the bottom of uncoated culture flasks [15]. At the end of the pre-plating time, neurons were floating in the incubation media and could be harvested by collecting the media. Satellite cells remained attached to the flask, in which 5 ml of fresh complete culture medium were added. Satellite cells were then incubated for a week, during which the medium was replaced twice, until reaching almost complete confluence. At this time, cells were detached from the flask by a 5-min 0.05% trypsin-EDTA (Biochrom) treatment at 37°C, resuspended in fresh complete culture medium, and plated at a density of 400,000 cells/ml using 96 well plates (100 μl/well) for functional experiments, 24 well plates (500 μl/well) for the preparation of conditioned media and 6 well plates (2 ml/well) for COX mRNA and protein analysis, respectively. Finally, for immunocytochemistry studies, cells were seeded on glass coverslips.

Neurons collected from the flask were plated at a density of 100,000 cells/well in 24-well tissue culture plates, previously coated with poli-D-lysine (40 μg/ml; MW 70,000–130,000, Sigma). The incubation volume was 1 ml/well of complete culture medium (see above), enriched with 50 ng/ml of 2.5 S murine nerve growth factor (Alexis Biochemicals). The culture medium was changed within 24 h from seeding, and 20 μM cytosine arabinoside was added to further reduce non-neuronal cell growth [15]. All experiments were carried out 6–7 days after dissection, when cells reached complete maturation [15].

### Experimental procedures

In preliminary experiments we characterized the activation of satellite cells under different pro-inflammatory stimuli. Cells were incubated for various times in complete culture medium with or without the addition of 10 ng/ml IL-1β or 100 μM DETA/NO. At the end of the experiments, media were collected and stored at -35°C until the day of the radioimmunological assessment of PGE2 content. For the analysis of COX expression, cells were either lysed in TRIZOL for mRNA analysis or in RIPA buffer (composition below) for protein analysis. Lysates were stored at -80°C until further processed.

To explore the molecular mechanisms underlying NO effects, cells were activated for 6 h with 100 μM DETA/NO alone or in presence of the soluble guanilyl cyclase inhibitor, NS2028 (1–1000 nM), or they were treated with 8-Br-cGMP (1–100 μM).

Conditioned media from activated satellite cells was generated following a more complex protocol intended to remove from the media the proinflammatory stimuli (IL-1β or DETA/NO) to avoid direct effect of these agents on neurons. Briefly, satellite cells were incubated in presence of 10 ng/ml IL-1β or 100 μM DETA/NO for various times, then this medium was removed and replaced with complete culture medium. After a 24-h conditioning period, this medium was collected and used in part to assess PGE2 release (results are shown in Figure 7), and in part was stored at -80°C until the experiments on neurons were performed. In the latter paradigm, conditioned media from satellite cells activated for 4 h with IL-1β or 6 h with DETA/NO were used. In preliminary experiments we tested the effects of conditioned media generated from resting satellite cells (100%) or satellite cell activated with IL-1β (dilutions ranging between 12.5% and 100%) on basal CGRP release from trigeminal neurons (30 min incubation), and we did not observe any significant effect. Therefore, neuronal cultures were pre-exposed to 50% conditioned media generated by activated satellite cells for 30 min and then further stimulated by 10 min incubation with a well characterized depolarizing stimulus, 100 nM capsaicin [15], in order to study the possible effects of glial factors on neuronal activation. At the end of these experiments, the medium was collected and stored at -35°C until the assessment of CGRP by a specific radioimmunoassay (RIA).

### PGE2 and CGRP radioimmunoassays

PGE2 was measured by RIA as previously described in detail [46]. Incubation mixtures of 1.5 ml were prepared in disposable plastic tubes. Culture derived media samples (100 μl) were diluted to 250 μl with 0.025 M phosphate buffer (pH 7.5) and mixed with [^3^H]PGE2 (2,500–3,000 cpm). Antiserum reactive against PGE2 (kindly provided by Prof. G. Ciabattoni) was added with sufficient phosphate buffer to achieve a final dilution of 1:120.000 in a total incubation volume of 1.5 ml. A standard curve was generated with duplicate aliquots of PGE2 at 2–400 pg/tube. After 24 h, free PGE2 was removed with activated charcoal (Sigma). Supernatants (containing antibody-bound PGE2) were combined with 10 ml liquid scintillation fluid, and radioactivity was measured by liquid scintillation counting. The detection limit of the assay was 2 pg/tube and the EC50 40 pg/tube. The intra- and inter-assay variability was 5 and 10% respectively.

CGRP release was measured by a specific RIA technique validated in our laboratory and previously described in detail [15]. Briefly, a buffer containing 10 mM sodium phosphate, 154 mM NaCl, 25 mM ethylene diamine tetraacetic acid (EDTA), 0.01% thimerosal, 0.5% BSA and 0.03% Tween 20, pH 7.2 was used. The RIA was performed as follows: 100 μl of sample or standard solution was diluted 3-fold into RIA buffer containing anti-hα-CGRP (kindly provided by Prof. D. Currò) at a final dilution of 1:120,000. After 24 h at 4°C, 100 μl of [^125^I]-hα-CGRP (6000 cpm/tube) was added and the incubation continued at 4°C for 48 h. Separation of free from bound α-CGRP was achieved by adding anti-rabbit goat serum (at final dilution 1:200) and 500 μl of 6.6% polyethylene glycol solution in 5 mM phosphate buffer, pH 7.4. After 2 h at 4°C, the tubes were centrifuged at 3000 × g for 30 min at 4°C, and the pellet counted in a γ-counter. The standard curve ranged from 1.95 to 1000 pg/tube of rα-CGRP. Each sample and standard was assayed in duplicate. The mean IC50 of the standard curve was 50.8 ± 2.0 pg/tube (*n *= 10) and non-specific binding of the labeled ligand was not different from the background of the γ-counter. The intra-assay (*n *= 6) and inter-assay (*n *= 10) coefficients of variation were, respectively, 1.27% and ± 0.95% at the lowest (1.95 pg/tube) and 13.7% and ± 12.6% at the highest (1000 pg/tube) levels of standard. The detection limit was 19.5 pg/ml.

### mRNA analysis

Total cytoplasmic RNA was extracted from satellite cells using TRIZOL (Invitrogen). RNA concentration was measured using the Quant-iT™ RiboGreen^® ^RNA Assay Kit (Invitrogen). In each assay, a standard curve in the range of 0–100 ng RNA was run using 16S and 23S ribosomal RNA (rRNA) from E. coli as standard. Aliquots (1 μg) of RNA were converted to cDNA using random hexamer primers. Quantitative changes in mRNA levels were estimated by real time PCR (Q-PCR) using the following cycling conditions: 35 cycles of denaturation at 95°C for 30 sec; annealing at 59°C for 60 sec; and extension at 72°C for 60 sec; using the Brilliant SYBR Green QPCR Master Mix 2× (Stratagene, La Jolla, CA). PCR reactions were carried out in a 20 μL reaction volume in a MX3000P real time PCR machine (Stratagene). The primers used for COX2 were: 677F (5'-GCA TTC TTT GCC CAG CAC TTC ACT-3'), and 774R (5'-TTT AAG TCC ACT CCA TGG CCC AGT-3'), which yield a 98 bp product. The primers used for COX1 detection were: 154F (5'-CCT CAC CAG TCA ATC CCT GT-3'), and 384R (5'-AGG TGG CAT TCA CAA ACT CC-3'), which yield a 231 base pair (bp) product. The primers used for GAPDH were: 1505F (5'-TCC CAG AGC TGA ACG GGA AGC TCA GTG-3'), e 1818R (5'-TGG AGG CCA TGT AGG CCA TGA GGT CCA-3'), which yield a 314 bp product. Relative mRNA concentrations were calculated from the take-off point of reactions (threshold cycle, Ct) using the comparative quantitation method performed by Stratagene software and based upon the -ΔΔCt method [47]. This analysis approximates a given sample's target mRNA (e.g., COX2) level relative to the mean of the target mRNA levels in untreated controls ("calibrator" value), thus permitting statistical analysis of deviation from the mean even among the controls. Ct values for GAPDH expression served as a normalizing signal. In each assay, the PCR efficiency was also calculated using serial dilution of one experimental sample; efficiency values between 94–98% were found for each primer set and taken into account for the comparative quantitation analysis. At the end of Q-PCR, the products were separated by electrophoresis through 2% agarose gels containing 0.1 μg/ml ethidium bromide to ensure the product was the correct size.

### Western immunoblot

Cultures were washed twice in ice-cold PBS and lysed in RIPA buffer (1 mM EDTA, 150 mM NaCl, 1% igepal, 0.1% SDS, 0.5% sodium deoxycholate, 50 mM Tris-HCl, pH 8.0) containing protease inhibitor cocktail diluted 1:250 (Sigma). The protein content in each sample was determined by Bradford's method using bovine serum albumin as standard. A 10-μg aliquot of protein was mixed 1:3 with 3× gel sample buffer (150 mM Tris-HCl pH 6.8, 7.5% SDS, 45% glycerol, 7.5% of bromophenol blue, 15% β-mercaptoethanol), boiled for 5 min, and separated through 10% polyacrylamide SDS gels. Apparent molecular weights were estimated by comparison to colored molecular weight markers (Biorad; Hercules, CA). After electrophoresis, proteins were transferred to polyvinylidene difluoride membranes by semi-dry electrophoretic transfer. The membranes were blocked with 10% (w/v) low-fat milk in TBST (10 mM Tris, 150 mM NaCl, 0.1% Tween-20, pH 7.6) for 1 h at room temperature, and incubated in the presence of anti-COX2 antibody (at 1:1,000 dilution) overnight with gentle shaking at 4°C. The primary antibody was removed, membranes washed three times in TBST, and further incubated for 1 h at room temperature in the presence of anti-rabbit IgG-HRP secondary antibody, diluted 1:15,000. Following three washes in TBST, bands were visualized by incubation in ECL reagents for 5 min and exposure to X-ray film for 1 min. The same membranes were washed 3 times in TBST, blocked with 10% (w/v) low-fat milk in TBST for 1 h at room temperature and used for β-actin immunoblot (incubating with anti-β-actin antibody at 1:1000 dilution, overnight with gentle shaking at 4°C and anti-mouse IgG-HRP secondary antibody, diluted 1:8000). Band intensities were determined using ImageJ software (National Institutes of Heatlh) from autoradiographs obtained from the minimum exposure time that allow band detection, and background intensities (determined from an equal-sized area of the film immediately above the band of interest) were subtracted [48].

### Immunocytofluorescence

For immunocytofluorescent characterization of rat trigeminal satellite cell cultures, cells were plated at a density of 5000 cells/well on coated glass coverslips and kept in culture for 3–4 days. At this time, cultures were washed three times with PBS containing Ca^2+ ^and Mg^2+ ^(PBS-w) and fixed in 4% paraformaldehyde for 20 min. After fixation, cultures were washed twice with PBS-w and blocked with 0.5% BSA in PBS-w for 30 min. Primary antibodies were diluted (1:1000 for mouse monoclonal anti-glial fibrillary acidic protein, GFAP, Chemicon – Millipore Corporation, Temecula, US; 1:500 for rabbit polyclonal anti-glutamine synthase, GS, Sigma) in PBS-w containing 0.1% BSA and 0.2% Triton X100 and applied to cultures overnight at 4°C. Secondary antibodies were horse anti-mouse IgG conjugated to fluorescein and goat anti-rabbit IgG conjugated to Texas Red (Vector Laboratories, Burlingame CA). Secondary antibodies were incubated for 1 h at 37°C and diluted 1:200 in PBS-w in 0.1% BSA. Coverslips were mounted using Vectashield mounting media. Images were obtained on a Nikon Eclipse TE300 inverted fluorescence microscope equipped with a Cool SNAP professional digital camera and LUCIA-G/F imaging software.

### Statistical analysis

All data are presented as mean ± SEM, unless otherwise stated. Significant differences between groups were assessed by one-way or two-way analysis of variance (ANOVA) followed by Bonferroni's *post-hoc *test. All data were analysed using a PrismTM computer program (GraphPad, San Diego CA, USA).

## Competing interests

The authors declare that they have no competing interests.

## Authors' contributions

AC contributed to experimental design, carried out the majority of the experiments and drafted the manuscript; ADC helped with the cell culture experiments and carried out immunocytochemistry and CGRP radioiummunoassays, contributed to the manuscript editing; LL carried out QPCR analysis and Western Immunoblot; GT carried out the PGE2 radioimmunoassays; PN contributed to critically revise the manuscript; and CDR supervised the research project, wrote the manuscript and approved the manuscript in the final form. All the authors read and approved the final manuscript.
